# Design of MEMS Pressure Sensor Anti-Interference System Based on Filtering and PID Compensation

**DOI:** 10.3390/s24175765

**Published:** 2024-09-05

**Authors:** Baojie Li, Guiling Sun, Haicheng Zhang, Liang Dong, Yunlong Kong

**Affiliations:** College of Electronic Information and Optical Engineering, Nankai University, Tianjin 300350, China; libj@mail.nankai.edu.cn (B.L.); zhc@mail.nankai.edu.cn (H.Z.); dliang@mail.nankai.edu.cn (L.D.); kongyl@mail.nankai.edu.cn (Y.K.)

**Keywords:** MEMS pressure sensor, PID algorithm, least squares, temperature drift, static characteristics

## Abstract

Due to the inherent temperature drift and lack of static stability in traditional pressure sensors, which make it difficult for them to meet the increasing demands of various industries, this paper designs a new system. The proposed system integrates temperature measurement and regulation circuits, signal processing, and communication circuits to accurately acquire and transmit pressure sensor data. The system designs a filtering algorithm to filter the original data and develops a data-fitting operation to achieve error compensation of the static characteristics. In order to eliminate the temperature drift problem of the sensor system, the system also adopts an improved PID thermostatic control algorithm to compensate for the temperature drift. Finally, it can also transmit the processed pressure data remotely. The experimental results show that the nonlinear error at 50 °C is reduced from the initial 1.82% to 0.24%; the hysteresis error is significantly reduced from 1.23% to 0.046%; and the repeatability error control is reduced from 3.79% to 0.89%. By compensating for thermal drift, the system’s thermal sensitivity drift coefficient is reduced by 74.67%, the thermal zero drift coefficient is reduced by 66.24%, and the wireless communication range is up to 1km. The above significant optimization results fully validate the high accuracy and stability of the system, which is perfectly suited for demanding pressure measurement applications.

## 1. Introduction

Sensors play a vital role in integrating the physical and digital domains by converting physical phenomena into digital signals. As the semiconductor industry enters a period of rapid development, piezoresistive sensors are widely used in key areas such as engine management systems, airbag control systems, and tire pressure monitoring systems. Their market size continues to expand, and their market share grows [1]. Although pressure sensors have many applications in various industries, some things could still be improved. One of the significant challenges in developing piezoresistive pressure sensors is the sensitivity of the piezoresistive coefficient of the semiconductor material to temperature changes. This inherent temperature dependence introduces thermal zero drift (the baseline output of the sensor varies with the ambient temperature) and thermal sensitivity drift (change in the gain factor of the sensor due to temperature fluctuations) in the sensor output, posing a significant barrier to achieving accurate measurements [2,3]. In addition, achieving higher accuracy static characteristics is a current challenge. These static characteristics include nonlinear (the degree of linearity of the sensor input and output curves), hysteresis (the degree of linearity of the sensor input and output curves), and repeatability (the degree of consistency of the sensor output when the same input is measured multiple times under the same conditions) [4,5]. Together, these factors affect the reliability and accuracy of the measurements, which in turn affects the system’s performance using the sensor. Therefore, it is critical to develop a robust system that can address these issues and improve the performance metrics of the pressure sensor. Such a system would mitigate the effects of temperature drift and improve the accuracy of the static characteristics, thereby improving the overall accuracy and reliability of the sensor measurements [6].

Recent research on piezoresistive pressure sensors mainly focuses on sensor nonlinear, hysteresis, repeatability, and temperature drift [7,8,9,10]. Various correction and compensation techniques have been developed and applied to mitigate these issues. Common approaches include universal algorithms and methods such as the least-squares method, Backpropagation Neural Network (BPNN), Newton’s algorithm, spline interpolation, and hardware-based compensation techniques [4,11]. The error of piezoresistive sensors can be improved by using new materials and structural innovations; literature [12] investigated a Silicon-On-Insulator (SOI)-based Micro Electro Mechanical System (MEMS) piezoresistive pressure sensor and optimized the positions and thickness of the pressure-sensitive elements in the piezoresistive sensor, which effectively improved the nonlinear error, hysteresis error, and repeatability error. Literature [13] investigated a wide range and high repeatability MEMS pressure sensor based on graphene array structure, and the sealing of the sensor was accomplished by using Au/Sn eutectic bonding technology, which improved the pressure sensor’s nonlinear error and hysteresis error. Although using MEMS structures can improve the static error of pressure sensors, the error due to temperature drift is reduced purely by structural design. In addition, unique MEMS structures are complex and costly to mass produce, and there are also challenges in signal processing, environmental adaptability, and long-term stability. In addition to using MEMS structures for pressure sensor performance optimization, literature [14] proposed a method of temperature compensation for MEMS pressure sensors using a thermistor network, which significantly reduces the thermal sensitivity drift compared with the traditional hardware compensation method. However, the technique reduces the thermal sensitivity drift but sacrifices a part of the output signal (from 58 mV to 32 mV); literature [15] proposed a MEMS composite sensor based on a stress-sensitive aluminum–silicon hybrid structure and applied the Elman neural network—optimized based on ant colony algorithm—to fuse the differential output data to eliminate the temperature drift error further. Literature [16] proposed a temperature compensation model based on particle swarm optimization radial basis function (RBF) neural network and least squares fusion to effectively suppress the temperature drift effect of silicon piezoresistive pressure sensors. The software compensation in the above studies requires accurate temperature data input. However, none of the researchers performed the relevant filtering process, and the algorithms used require a large amount of data for training and optimization themselves, as well as more arithmetic resources, and only apply to some applications. Some studies use thermostatic operation for temperature drift compensation [17]. However, the study used a thermostat oven for thermostatic operation, which requires too much power, and the system size needs to be bigger, making it unsuitable for small application scenarios.

This study has significantly improved static error when comparing and analyzing the errors listed in Table 1. The nonlinear error in this study is 0.24%, which indicates that this study maintained a linear relationship better when processing the measurement data, thus improving the accuracy of the measurement results. The hysteresis error is 0.046%, the lowest of all the studies, indicating that the measurement system in this study has a very high degree of consistency during forward and reverse measurements, which is especially important for application scenarios that require precise control. The repeatability error is 0.89%, which is not the lowest but still within the acceptable range, indicating that the measurements in this study have good consistency. In addition, this study introduced a small heating film and Proportion-Integration-Differentiation (PID) algorithm for temperature compensation, and this technique significantly improved the thermal stability of the system. The values of thermal sensitivity drift and thermal zero drift are −0.0016% and −0.002%, respectively, and these extremely low drift coefficients indicate that the measurement system in this study remains highly stable under temperature variations.

Summarizing the conclusions related to temperature drift and static error methods in the above studies and inspired by the findings of the above studies, this paper designs and implements a pressure sensor automatic monitoring and regulation system, which is based on an STM32L432 microcontroller and an MEMS piezoresistive pressure sensor, with the following main design innovations:

1. Static characteristic compensation: the recursive filtering algorithm is used to effectively filter out the noise in the sensor’s original acquisition signal, and the sensor’s static characteristic is compensated for the error by the least squares method.
2. Temperature drift compensation: an improved PID thermostatic control algorithm is used to compensate for the temperature drift of the sensor, which significantly improves the stability of the sensor’s performance at different temperatures.

## 2. Materials and Methods

### 2.1. General Design

**Figure 1:**
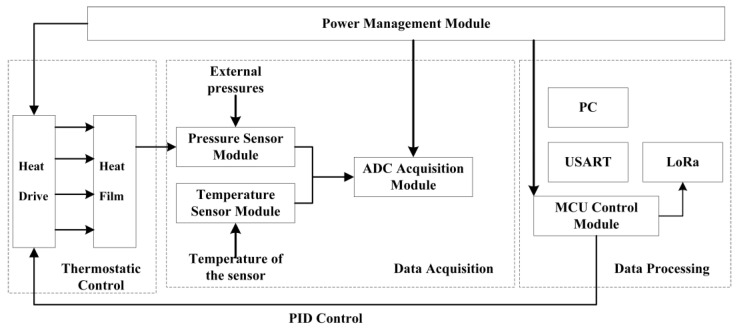
Overall design framework for pressure measurement systems.

The sensor pressure monitoring and regulation system collects optimized pressure data information, enabling temperature and static error compensation functions. Pressure data can be transferred to a personal computer via a serial port or remotely via a LoRa module. The system consists of a microcontroller, a MEMS pressure sensor, a temperature sensor, a Long Range (LoRa) transmission module, a heating resistor, a driver module, and a power management module; the framework is shown in Figure 1. The three most important parts of the system are data acquisition, data processing, and thermostatic control. In general, the data acquisition part takes the MEMS pressure sensor as the core, and the signal acquisition circuit collects the signals from the pressure sensor and transmits them to the microcontroller for processing; the data processing part transmits and displays the collected pressure and temperature signals through the Universal Synchronous/Asynchronous Receiver/Transmitter (USART) and LoRa protocols; in the thermostatic control part, the temperature sensor is placed between the pressure sensor and the heating membrane, which can measure the temperature of the sensor chip in real-time. The power amplifier driver circuit activates the heating membrane’s heating by outputting a Pulse Width Modulation (PWM) wave.

The microcontroller of the system selects STM32L432KBU6, the L4 series energy efficiency is better than the first introduced L0, L1 series, the maximum operating frequency of 80 MHz, the operating power consumption of 31 μA/MHz, and the chip for the UQFN32 package, which contributes to the miniaturization of the node design to meet the node function requirements; the communication part of the selection of the SX1268 module applicable to the CN470 band E22-400M22S, compared with the SX1268 module E22-400M22S, the communication part of the communication part applies to the CN470 frequency band. 400M22S, compared with SX1276, SX1278, and SX1268, has a lower receive current and higher transmit power; the module integrates a low noise amplifier and power amplifier, and the highest receive sensitivity can reach −148 dBm when LoRa modulation the maximum transmit power can reach +22 dBm [18]. DC-DC mode receive current is 4.6 mA, the highest can provide 170 dB link budget; the power supply module chooses TPS6305 for monitoring node system main power supply, lift voltage topology can be in the 6–8.4 V lithium battery voltage range to produce a stable 5 V 1 A output, and the efficiency is maintained at more than 90%. TPS22917 to achieve the power supply of each part of the independent control does not work when disconnecting the module power to reduce power consumption.

The MEMS pressure sensor consists of a Wheatstone bridge with two sets of pins, one for accessing the current source for the power supply and the other for outputting a voltage signal to reflect the pressure change. The digital-to-analog converter module adopts the AD7794 signal acquisition chip, which has 24-bit resolution and six differential analog inputs, supports up to 128 times signal amplification, built-in low-noise instrumentation amplifier, as well as a precision internal bandgap reference power supply. The temperature sensor utilizes a PT1000 platinum Resistance Temperature Detector (RTD), which has a precise function between resistance and temperature, ensuring accurate and sensitive temperature measurements. The PT1000 has an extremely wide operating temperature range of −200 °C to 850 °C, making it suitable for various ambient conditions. In order to adapt to the different temperature conditions, a polyimide heating diaphragm has been chosen as the heating device. These heating foils are thin, lightweight, and easy to mount on the sensor’s printed circuit board package. In order to satisfy the efficiency of the heating film and the need for a larger operating power to drive the chip, TB6612FNG was chosen, which has an operating temperature of −20 °C~85 °C, covering the constant temperature operating range of the sensor set in this paper.

### 2.2. MEMS Pressure Sensor Design and Characterization

#### 2.2.1. MEMS Pressure Sensor Design

**Figure 2:**
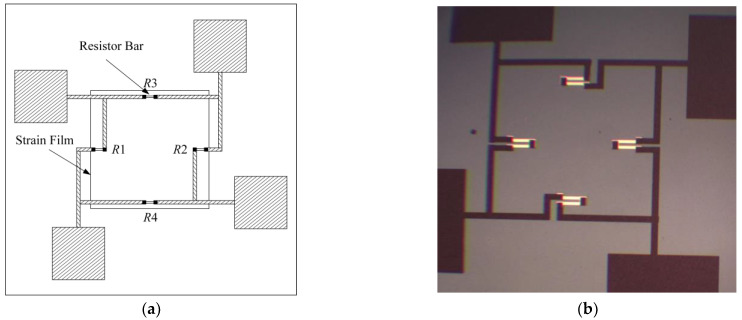
(**a**) MEMS pressure resistor structure schematic; (**b**) MEMS pressure sensor.

MEMS piezoresistive pressure sensors utilize the piezoresistive effect of silicon to convert the external pressure into a resistance change, which is converted into a voltage signal through a Wheatstone bridge circuit, which is a circuit consisting of four resistors that are connected diagonally two by two to form a closed loop [19]. In MEMS pressure sensors, two of the four Wheatstone bridge resistors are piezo resistors located in the strain film of the sensor, whose structure is shown in Figure 2a. When the sensor is pressurized, the strain film deforms, resulting in a change in the resistance of the piezoresistor, and this change breaks the balance of the bridge, resulting in a voltage difference across the diagonal of the bridge. When the temperature changes, the resistance value also changes at any time.

The raised sensor piezo resistor structure is fabricated using SOI silicon wafers in this design, and the four raised L-shaped peninsula structures become the sensor’s key part. Figure 2b shows a MEMS pressure sensor. When pressure is applied externally, the sensor diaphragm deforms, and resistors R1 and R2 resistors increase. In contrast, the resistance of R3 and R4 decreases, which is practically the same amount of change in resistance. The relationship between the output voltage of the Wheatstone bridge and the resistance can be derived from Equation (1): the sensor’s output voltage is directly proportional to the change in resistance. The amount of change in resistance is directly proportional to the applied pressure, so the magnitude of the applied pressure can be derived from the amount of change in the output voltage of the sensor [20].

(1) $$V_{\mathrm{out}}=V_{\mathrm{in}}\left(\frac{\left(R_{1}+\Delta R_{1}\right)\left(R_{2}+\Delta R_{2}\right)-\left(R_{3}-\Delta R_{3}\right)\left(R_{4}-\Delta R_{4}\right)}{\left(R_{1}+R_{2}+\Delta R_{1}-\Delta R_{2}\right)\left(R_{3}+R_{4}+\Delta R_{3}-\Delta R_{4}\right)}\right)$$

#### 2.2.2. Static Characterization of MEMS Pressure Sensors

The static characteristics of a pressure transducer refer to the ability of the transducer to respond to a specific pressure change under stable operating conditions without the influence of external dynamic factors. The parameters of the static characteristic play a decisive role in characterizing the sensor’s performance. The following are three important errors in static characteristics: nonlinear error, hysteresis error, and repeatability error.

1. Nonlinear Error

Nonlinear error is used to show the linearity of the sensor’s input and output curves and can be expressed by Equation (2) [21]. Ideally, the sensor’s output should be linear with the input. However, due to various disturbances in the actual situation and the non-ideal characteristics of the sensor itself, there is a deviation between the actual output and the ideal straight line.

(2) $$e_{NL}=\frac{\Delta_{\max}}{V_{\mathrm{FS}}}\times 100\%$$

The $\Delta_{\max}$ is the maximum deviation error and $V_{\mathrm{FS}}$ is the full-scale output.

2. Hysteresis Error

When the input signal of the sensor is increased from zero to the maximum value and then returned to zero, the path of the output signal is not consistent; this phenomenon is called hysteresis, which can be expressed by Equation (3) [22]. The hysteresis error reflects the speed and accuracy of the sensor’s response to changes in the input.

(3) $$e_{H}=\frac{\Delta H_{\max}}{Y_{\mathrm{FS}}}\times 100\%$$

The $\Delta H_{\max}$ represents the maximum offset difference between the forward and reverse stroke output curves.

3. Repeatability Error

The repeatability error reflects the stability and reliability of the sensor, which can be expressed by Equation (4) [23].

(4) $$e_{R}=\frac{{\Delta R}_{\max}}{Y_{\mathrm{FS}}}\times 100\%$$

The ${\Delta R}_{\max}$ is the maximum difference between the outputs of multiple measurements.

#### 2.2.3. Thermal Zero Drift and Thermal Sensitivity Drift

**Figure 3:**
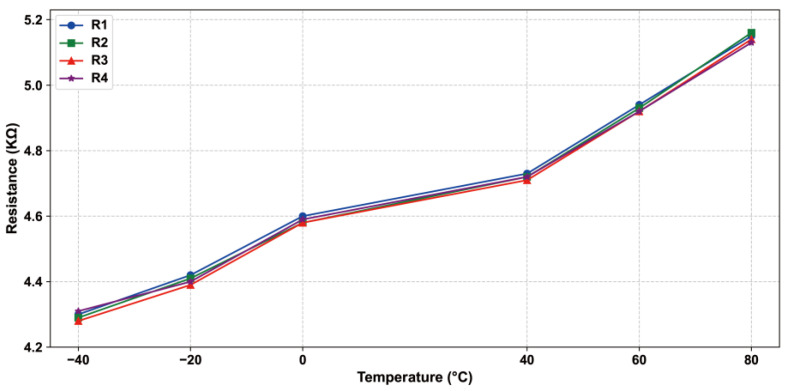
Trend of Wheatstone bridge with temperature.

Under ideal conditions, when the resistors are not affected by strain, their resistance values are equal, the bridge is balanced, and the output signal should theoretically be zero. However, in practice, the resistance of the Wheatstone Bridge will be affected by temperature changes, as shown in Figure 3. Then, the sensor’s output signal will be due to temperature changes and offset [24]. Equation (5) describes the sensor, without external forces, the output signal with the temperature change and the resulting deviation, the thermal zero drift coefficient.

(5) $$k_{0}=\frac{V_{0}\left(T_{2}\right)-V_{0}\left(T_{1}\right)}{V_{\mathrm{FS}}\left(T_{1}\right)\left(T_{2}-T_{1}\right)}\times 100{\%\mathrm{FS}/}^{\circ }C$$

$V_{\mathrm{FS}}$ is the full-scale output voltage at room temperature; $V_{0}\left(T_{2}\right)$ is the zero-point output voltage at temperature T2; $V_{0}\left(T_{1}\right)$ is the zero-point output voltage at room temperature T1.

Temperature fluctuations lead to drift in the sensor’s zero output and cause changes in the sensor’s sensitivity. The material properties mainly cause this drift; in the temperature change, doped silicon internal carrier mobility will change, resulting in piezoresistive effect and resistivity changes, affecting the sensor’s output signal. At the same time, due to the different coefficients of thermal expansion of the pressure-sensitive element and the strain film material, thermal stresses are generated between the two materials when the temperature changes. This stress causes strain in the pressure-sensitive element, leading to a shift in sensitivity. In order to quantify this temperature effect on sensitivity, Equation (6) is introduced to represent the thermal sensitivity drift coefficient [25]. A smaller coefficient indicates that the sensor’s output signal is less sensitive to temperature changes, which is essential to maintain the sensor’s measurement accuracy under different temperature conditions.

(6) $$k_{s}=\frac{V_{\mathrm{FS}}\left(T_{2}\right)-V_{\mathrm{FS}}\left(T_{1}\right)}{V_{\mathrm{FS}}\left(T_{1}\right)\left(T_{2}-T_{1}\right)}\times 100{\%\mathrm{FS}/}^{\circ }C$$

### 2.3. Experimental Program

#### 2.3.1. Overall Software Program

**Figure 4:**
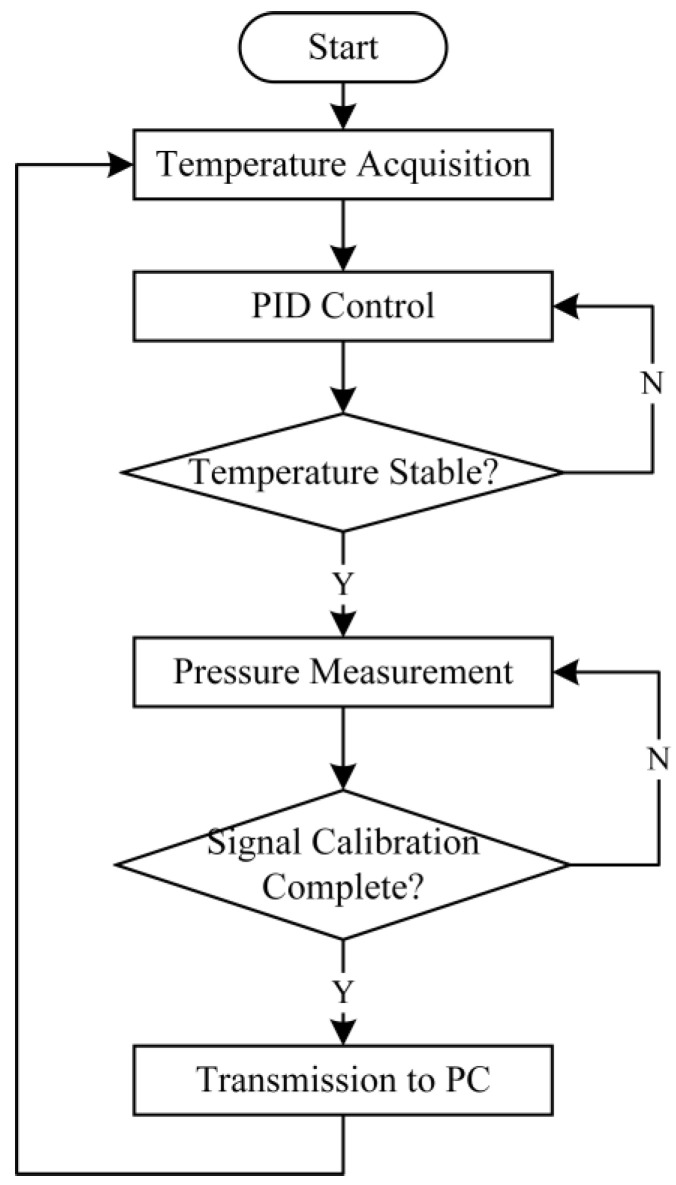
Overall system software operation program.

The algorithm flow of the system to collect the pressure sensor information and regulate it is shown in Figure 4. Firstly, the temperature data is collected, and the heating circuit and constant-temperature PID algorithm recognize the temperature compensation. When the temperature is stabilized to the target value, the pressure sensor information is collected, filtered, and optimized by recursive filtering and least squares. Then, the static characteristics of the sensor and the temperature drift performance after thermostatic compensation are tested and transmitted remotely using the LoRa module.

#### 2.3.2. Filtering Algorithm Design

Due to the tiny size of the MEMS sensor, it is very sensitive to environmental noise and interference, resulting in the output signal containing a large random noise component; in order to optimize the accuracy of the data, it is first necessary to use a filtering algorithm to denoise the original data. After an in-depth analysis of the experimental data, a recursive filtering method was selected for processing the pressure sensor’s output signal data, as it is well-suited for this application. The recursive filtering method is suitable for processing signals with serious noise interference [26]. This filtering method through the sampling data weighted summing operation so that the random interference signals offset each other, obtain stable and reliable output data, and improve the system’s anti-interference ability. The sampled value and the current sampled value calculated by the filtering formula can be expressed by Equation (7).

(7) $$b_{i}=\frac{a_{i}}{n}+\alpha \frac{a_{i-1}}{n}+\alpha^{2}\frac{a_{i-2}}{n}+\ldots +\alpha^{i-1}\frac{a_{1}}{n}(i\geq 2),b_{1}=a_{1}$$

where $\alpha =\frac{n-1}{n}$, $a_{j}$, *j* = 1, 2,…, *i* is the actual voltage value of the *j*th sample, $b_{i}$ is the voltage output after the recursive filtering process, *i* is the number of samples, $\frac{\alpha^{i-j}}{n}$ is the weighting coefficient, and the values of *i* and n are derived from experiments. According to Equation (7), it can be seen that each sampled data contributes to the output data, and the weighting coefficient determines the size of the contribution. For the value of *n*, the larger the value, the stronger the anti-interference ability of the output data, but at the same time, it will make the convergence speed of the operation slower, and the number of calculations will be increased accordingly. Through experimental verification, when *n* = 16 and the number of sampling operations is 72 times, the ideal error of the filtered output data is minimized.

**Figure 5:**
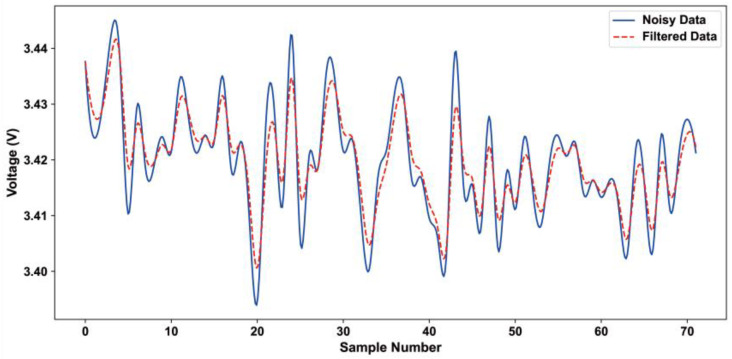
Comparison of pressure data collected before and after using recursive filtering method.

The recursive filtering method uses the random interference signal in all directions of the same nature, the use of weighted summation operation on the sampling data processing so that the interference signals offset each other to obtain stable and reliable output data; the whole system’s anti-jamming ability has been greatly improved. Table 2 shows the error characteristics of different filtering methods. It can be seen that, compared with the mean filtering method and median average filtering method, the recursive filtering method has good resistance to random interference and achieves satisfactory measurement results, producing an error of only 0.29%, which is significantly better than the other two methods. Figure 5 shows the output data comparison before and after recursive filtering processing.

#### 2.3.3. Least Squares Optimization

During the measurement process, it is found that the output signal of the MEMS piezoresistive pressure sensor is nonlinear, and the phenomena of hysteresis error and repeatability error also exist between the positive and negative strokes. In order to attenuate the influence of these errors on the sensor performance, this study adopts the least squares-based method to compensate for the static errors of MEMS pressure sensors. The Equation (8) [27,28] describing the output empirical curve of the pressure sensor using the least squares method is

(8) V=AP+B

$A=\frac{N\sum_{i=1}^{N}\left(P_{i}V_{i}\right)-\left(\sum_{i=1}^{N}P_{i}\right)\left(\sum_{i=1}^{N}V_{i}\right)}{N\sum_{i=1}^{N}P_{i}^{2}-{\left(\sum_{i=1}^{N}P_{i}\right)}^{2}}$, $B=\frac{\sum_{i=1}^{N}V_{i}-A\sum_{i=1}^{N}P_{i}}{N}$, *N* is the number of samples, $P_{i}$ is the pressure value of the sampling point, and $V_{i}$ is the value of the output data of the sensor sampling point.

#### 2.3.4. Improved PID Algorithm

Due to the material characteristics of the MEMS sensor, its resistance value will drift with the temperature change, resulting in measurement errors. In order to solve this problem, the system adopts a thermostatic control module based on the PID algorithm: STM32 processor through the PID algorithm to control the duty cycle of the PWM wave to achieve thermostatic compensation. The principle of the traditional PID algorithm is to adjust the results of the PID algorithm through the parameters and constant feedback applied to the system; when the actual output parameters of the system do not match the set value, the difference will be passed into the PID algorithm system, according to the PID algorithm to change the duty cycle parameter value, in order to control the chip temperature, so that the temperature parameters are gradually close to the system set to achieve the purpose of the thermostat [29].

**Figure 6:**
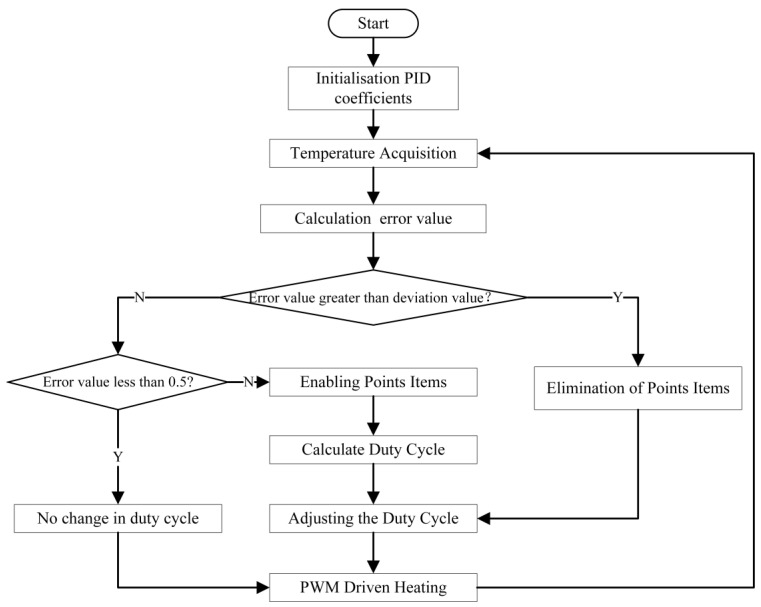
Improved PID thermostatic control flow.

In practical applications, temperature data sampling occurs at discrete intervals, and the collected data are discrete values. Additionally, the data has storage limitations, making older data less valuable for reference. Due to the integral action, significant errors can lead to system output overshoot, causing temperature oscillations around the target value. When system errors are small, relying solely on proportional and derivative control often fails to eliminate the error quickly, which affects the system’s response speed and accuracy [30,31]. Therefore, in this design, the main record of an integral part of the deviation value within a certain period, and to improve the system’s stability and response speed, the integral separation PID control algorithm is used. Equation (9) indicates that when the system error is large, the integral control part is canceled, and only the proportional and differential control are retained to avoid the overshoot caused by the integral action. When the error is small, the integral control is reintroduced to reduce the static error and improve the system stability [32,33]. The flowchart of the improved PID formulation can be represented in Figure 6.

(9) $$\Delta u(k)=K_{p}[e(k)-e(k-1)]+\beta K_{i}e(k)+K_{d}[e(k)-2e(k-1)+e(k-2)]$$

where e(k) is the error between the current temperature measured by the temperature sensor and the set temperature, Δu(k) represents the heating power and the strength of the system’s response to the current error. For temperature control systems, $K_{p}$ determines the strength of the system’s immediate response to temperature deviations. $K_{i}$ is the control system’s response to the accumulated error, which is important for eliminating long-term temperature deviations. The integral term is mainly used to eliminate the steady state error and maintain the temperature at the set point. $K_{d}$ is used to predict future error trends based on the rate of change in the current error. It reduces overshooting during thermoregulation caused by external perturbations or a control response that is too fast. β is the integral separation coefficient, β = 0 when the error is greater than the threshold, and β = 1 when the error is less than or equal to the threshold.

**Figure 7:**
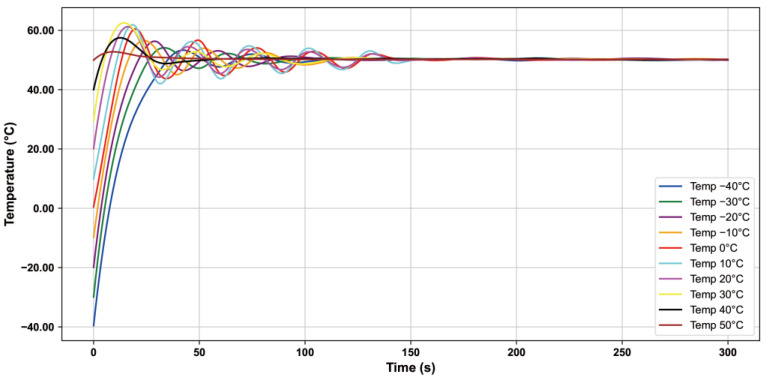
Temperature profiles of pressure sensors based on PID control.

In this paper, we use the heating pad and PID algorithm to control the constant temperature of the pressure sensor chip, record the temperature of the pressure sensor when the temperature of the temperature box is set to −40 °C~50 °C in 300 s, and record the temperature value once every 10 s. According to the test data, the corresponding temperature control curve is plotted as shown in Figure 7, and the results show that under different temperature environments, the heating pad fluctuates for some time and tends to stabilize in about 150 s. The chip temperature is maintained at about 50 °C.

## 3. Results

### 3.1. Experimental Environment

**Figure 8:**
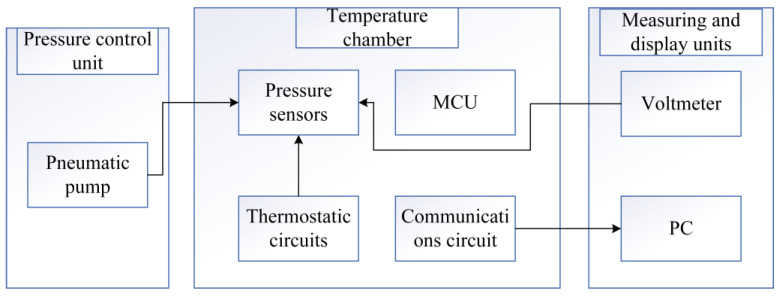
System test platform.

During the testing and validation phase, Figure 8 illustrates the schematic diagram of the system test platform, which includes a pressure pump, a temperature chamber simulating external temperature requirements, a pressure acquisition unit, and a host for displaying data. In the experiment, the pressure sensor is first sealed and then connected to the gas conduit of the pressure pump unit, which is subsequently placed inside the temperature test chamber. The pressure and temperature of the test chamber are adjusted, and pressure and temperature data from the sensor are read via a serial communication line.

To meet the experimental requirements, a vacuum pressure pump was utilized, capable of generating pressures ranging from 0 to 500 kPa with an accuracy of ±0.008%. Given that the measurement pressure range for this experiment is 0 to 300 kPa, the pressure pump is suitable for the measurement needs. The temperature chamber offers a temperature adjustment range from −90 °C to 150 °C, with a temperature change rate of 2 to 5 °C/min and temperature fluctuations not exceeding ±0.5 °C. This temperature chamber boasts excellent performance, featuring intelligent operational management, a user-friendly touchscreen interface, and a variety of data interfaces. It can be networked with devices such as personal computers, making it highly suitable for laboratory temperature testing experiments. The pressure signal measurement circuit board operates in conjunction with the host computer to measure the voltage output from the pressure sensor. This circuit board is capable of measuring both pressure and temperature signals, controlling the temperature of the pressure sensor, and transmitting the measurement results to the host computer.

**Figure 9:**
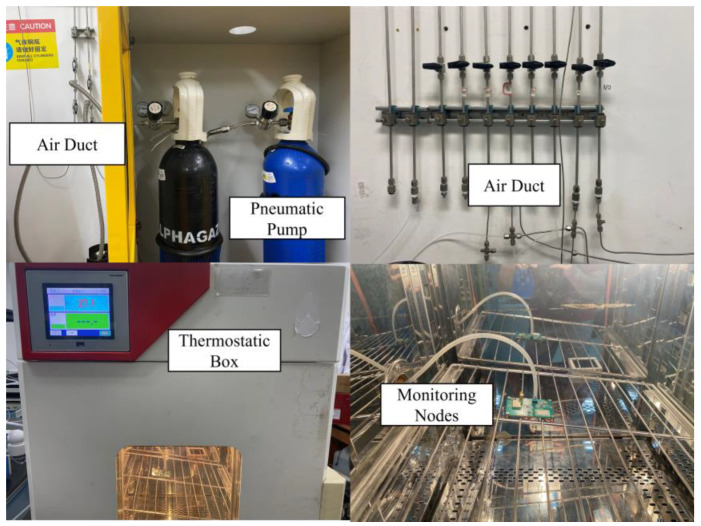
Experimental platforms.

**Figure 10:**
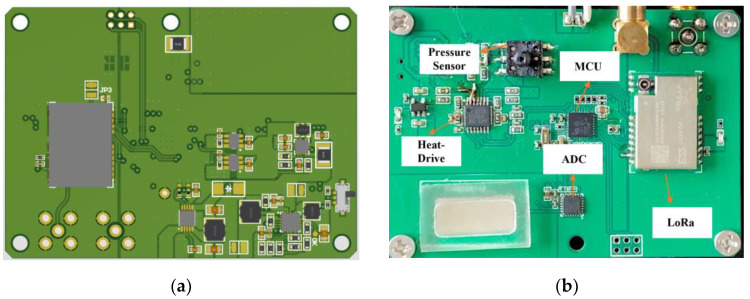
(**a**) The PCB of sensor pressure monitoring and regulation systems; (**b**) Pressure acquisition unit.

The actual experimental setup is illustrated in Figure 9. In the experiment, the MEMS piezoresistive pressure sensor measurement circuit is placed inside the temperature chamber and is connected to the pressure pump via a gas conduit. The chamber temperature is first set and allowed to stabilize. Subsequently, the pressure valve is adjusted to provide different pressure levels. The PC receives pressure and temperature data from the sensor measurement circuit through a serial communication line. After completing a set of measurements, the temperature of the test chamber is adjusted, and the procedure is repeated until all data points are collected. Figure 10a represents the monitoring terminal’s front-side PCB design; the specific configuration of the MEMS piezoresistive pressure sensor measurement circuit PCB is shown in Figure 10b.

### 3.2. Static Error Analysis

**Figure 11:**
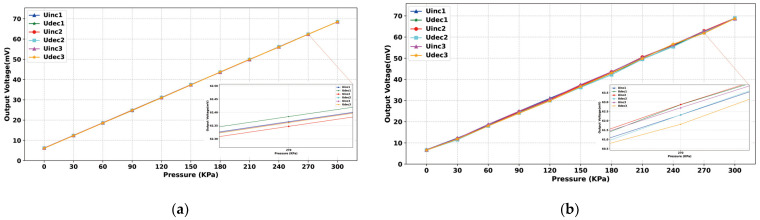
(**a**) Output voltage versus external pressure curve before fitting; (**b**) Output voltage versus external pressure curve after fitting.

**Figure 12:**
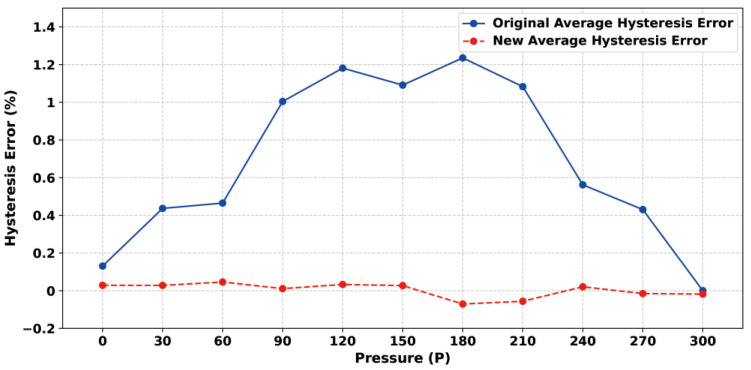
Pressure sensor hysteresis error test results.

**Figure 13:**
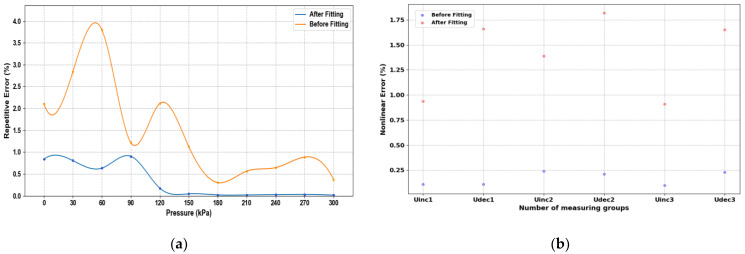
Pressure sensor repeatability error and nonlinear error test results: (**a**) Repeatability error; (**b**) Nonlinear error.

After the experimental platform was built, the static characteristics of the sensor were first measured and regulated, and the constant temperature was set at a higher level of 50 °C to meet the sensor chip and realize constant temperature control in various normal environments. The static characteristics of the sensor were obtained by changing the input pressure to obtain the output voltage value and extracting and calculating six sets of data, including three positive and three negative strokes. Figure 11a shows the input–output relationship before the least squares compensation, from which it can be seen that the output voltage of the sensor chip generally increases linearly with the increase in air pressure, and the calculated nonlinear error is 1.82% at maximum, the hysteresis error is 1.23% at maximum, and the repeatability error is 3.79% at maximum. During the measurement process, it is found that the output signal of the MEMS piezoresistive pressure sensor shows a certain nonlinear, and there are hysteresis errors and nonlinear errors between positive and negative strokes. In order to attenuate the impact of these static errors on the sensor’s performance, the data can be linearized using the least squares algorithm. Figure 11b shows the relationship between the input and output data after the least squares algorithm processing, showing that the positive and negative travel curves are closer after processing. Figure 12 represents the hysteresis error of the sensor before and after processing by the least squares algorithm, and Figure 13a represents the repeatability error before and after processing. Figure 13b represents the number of nonlinear errors before and after processing by the algorithm. It can be seen that after processing by the algorithm, the nonlinear error at 50 °C is reduced from the initial 1.82% to 0.24%, the hysteresis error is reduced from 1.23% to 0.046%, and the repeatability error control is reduced from 3.79% to 0.89%.

### 3.3. Temperature Drift Error Analysis

**Figure 14:**
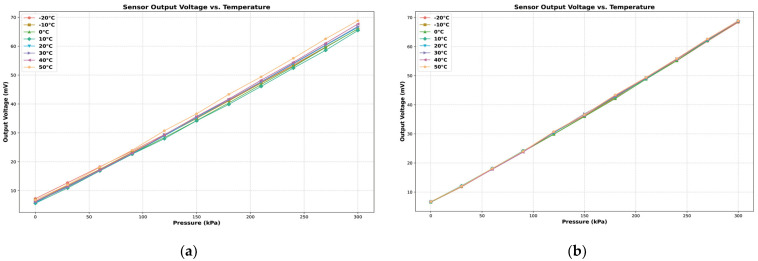
(**a**) Output curve after temperature compensation pressure sensor output voltage results at different temperatures without PID control; (**b**) Output curve after temperature compensation.

After addressing the static characteristic compensation, sensor temperature drift remained an issue. The same experimental platform was utilized to handle this problem. Without employing PID control for maintaining constant temperature, the temperature chamber was used to simulate various experimental temperature environments, while the pressure pump controlled the pressure environment for the sensor. Starting at 50 °C, the temperature was sequentially decreased in 10 °C increments, down to a minimum of −20 °C. The sensor’s output voltage was recorded under different pressure conditions at each temperature. Figure 14a illustrates the relationship between sensor output and temperature variations. A temperature of 50 °C was used as the reference temperature to analyze the sensor’s temperature drift characteristics. The thermal zero drift and temperature-induced thermal drift coefficients were calculated for each temperature point to quantify the sensor’s drift characteristics.

**Figure 15:**
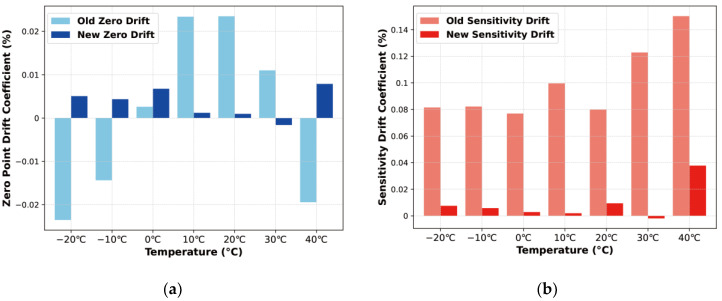
Thermal zero drift and thermal sensitivity drift before and after temperature compensation: (**a**) Comparison of thermal zero drift; (**b**) Comparison of thermal sensitivity drift.

To mitigate the measurement errors caused by temperature drift, this study implemented a heating pad with a PID algorithm to maintain the pressure sensor chip’s temperature at a constant 50 °C. Figure 14b presents the sensor’s output voltage versus pressure after temperature compensation. Continued temperature drift measurements were conducted following this temperature compensation approach. Figure 15a compares the thermal zero drift before and after temperature compensation, while Figure 15b compares the thermal sensitivity drift before and after. Additionally, Table 3 displays the STM32 control duty cycle for the heater driver pin outputs at various temperatures, maintaining a constant temperature of 50 °C.

Based on the data comparison, it is evident that utilizing 50 °C as the reference temperature significantly enhances the stability of the sensor’s output signal through thermostatic compensation. Specifically, the thermal zero drift improves from −0.0246% to 0.0237% FS/°C to −0.0016% to 0.0080% FS/°C following compensation. Similarly, the thermal sensitivity drift is reduced from 0.0762% to 0.150% FS/°C to −0.002% to 0.038% FS/°C. These improvements demonstrate that thermostatic control effectively mitigates temperature-induced variations, enhancing the sensor’s performance stability.

### 3.4. LoRa Transmission Distance Test

**Figure 16:**
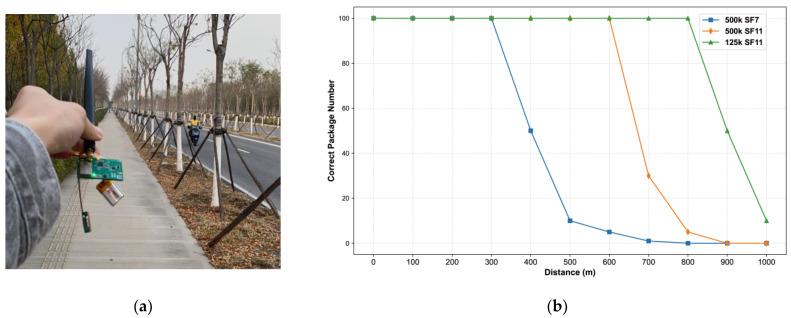
(**a**) Test scenario of LoRa module transmission distance in open field; (**b**) Test results of LoRa communication at different distances in open area.

To test the communication distance of a sensor node using LoRa, the E22-400M22S module was employed. LoRa frames were transmitted and received in a straight line along a suburban road, and the test scenario is shown in Figure 16a. The transmission power was set to +22 dBm, with a center frequency of 433 MHz, and the antenna height was maintained at 2 m above the ground. The LoRa parameters were configured as follows: BW 500 kHz, SF11, CR4/5; BW 500 kHz, SF7, CR4/5; and BW 125 kHz, SF11, CR4/5. These settings correspond to receive sensitivities of −117 dBm, −128.5 dBm, and −134.5 dBm, respectively [34,35]. For each set of parameters, 100 packets were transmitted every 100 m, and the number of correctly received packets was recorded to evaluate the communication performance. The communication distance and results for different LoRa parameters are shown in Figure 16b.

## 4. Conclusions

In response to the growing demand for high-performance pressure sensors, this paper presents a novel Sensor Interference Mitigation System designed to overcome the limitations of traditional pressure sensors, particularly temperature drift and static instability. The system integrates temperature regulation, signal processing, and communication circuits to enable precise pressure data acquisition and transmission.

A key innovation is the implementation of an enhanced PID control algorithm, effectively compensating for temperature drift. Additionally, a filtering algorithm improves data integrity by reducing noise and errors in sensor readings. Experimental validation shows significant performance improvements: nonlinear error at 50 °C was reduced from 1.82% to 0.24%, hysteresis error from 1.23% to 0.046%, and repeatability error from 3.79% to 0.89%. The thermal sensitivity drift and thermal zero drift coefficients were reduced by 74.67% and 66.24%, respectively. The system’s wireless communication achieved a transmission range of up to 1 km, highlighting its accuracy and stability for industrial applications.

Looking ahead, our goal is to significantly enhance the Sensor Interference Mitigation System by seamlessly integrating it with Internet of Things (IoT) technology. This strategic integration promises to deliver real-time monitoring and advanced data analytics, thereby elevating operational efficiency. Concurrently, we are committed to crafting adaptive algorithms powered by machine learning, which will dynamically refine temperature compensation. We are dedicated to the miniaturization of sensor components, aiming to produce more compact and cost-effective solutions. These concerted efforts are designed to substantially augment the capability and versatility of pressure-sensing technology, solidifying its position at the vanguard of contemporary industrial applications.

## Figures and Tables

**Table 1:** Comparison of pressure sensor performance errors in different studies.

References	Nonlinear Error (%)	Hysteresis Error (%)	Repeatability Error (%)	Thermal Sensitivity Drift (FSS/°C)	Thermal Zero Drift (FSS/°C)
Study 1 [11]	0.64	0.82	0.48	−0.375%	−0.017%
Study 2 [13]	/	2.112	4.067	/	/
Study 3 [14]	0.21	0.12	0.17	/	1.8%
Study 4 [15]	0.29	0.09	0.53	/	/
This Study	0.24	0.046	0.89	−0.0016%	−0.002%

**Table 2:** Error characteristics of different filtering methods.

Measured Value/V	Mean Value Filter/V	Median Value Filter/V	Recursive Filter/V	Mean Value Filtering Error/%	Median Filtering Error/%	Recursive Filtering Error/%
3.42	3.47	3.46	3.43	1.5	1.2	0.29

**Table 3:** Heater duty cycle data sheet at different initial temperature.

Temperature/°C	Duty Cycle%
−40	44.7
−30	40.3
−20	32.9
−10	27.6
0	24.4
10	18.4
20	13.2
30	8.0
40	2.2
50	0.05

## Data Availability

All data generated or analyzed during this study are included in this article.
